# Accumulation of somatic mutations leads to genetic mosaicism in cannabis

**DOI:** 10.1002/tpg2.20169

**Published:** 2021-11-22

**Authors:** Kristian Adamek, Andrew Maxwell Phineas Jones, Davoud Torkamaneh

**Affiliations:** ^1^ Dep. of Plant Agriculture Univ. of Guelph Guelph ON N1G 2W1 Canada; ^2^ Dép. de Phytologie Univ. Laval Québec QC G1V 0A6 Canada

## Abstract

Cannabis (*Cannabis sativa* L.) is typically propagated using stem cuttings taken from mother plants to produce genetically uniform propagules. However, producers anecdotally report that clonal lines deteriorate over time and eventually produce clones with less vigor and lower cannabinoid levels than the original mother plant. While the cause of this deterioration has not been investigated, one potential contributor is the accumulation of somatic mutations within the plant. To test this, we used deep sequencing of whole genomes (>50×) to compare the variability within an individual cannabis cultivar Honey Banana plant sampled at the bottom, middle, and top. We called over six million sequence variants based on a reference genome and found that the top had the most by a sizable amount. Comparing the variants among the samples uncovered that nearly 600,000 (34%) were unique to the top while the bottom only contained 148,000 (12%), and middle with 77,000 (9%) unique variants. Bioinformatics tools were used to identify mutations in critical cannabinoid–terpene biosynthesis pathways. While none were identified as high impact, four genes contained more than double the average level of nucleotide diversity (π) in or near the gene. Two genes code for essential enzymes required for the cannabinoid pathway while the other two are in the terpene pathways, demonstrating that mutations were accumulating within these pathways and could influence their function. Overall, a measurable number of intraplant genetic diversity was discovered that could impact long‐term genetic fidelity of clonal lines and potentially contribute to the observed decline in vigor and cannabinoid content.

## Core Ideas

- Whole‐genome sequencing reveals genetic diversity within a single cannabis plant.
- The genetic mosaicism hypothesis applies to cannabis.
- Highest number of unique variants were found from the top tissue sample.
- Replacing mother plants from middle region clones may minimize accumulating mutations.

**Abbreviations**
CBD: cannabidiol
GMH: genetic mosaicism hypothesis
LD: linkage disequilibrium
SNP: single‐nucleotide polymorphism
THC: tetrahydrocannabinol
WGS: whole‐genome sequencing.

## 1 INTRODUCTION

*Cannabis sativa* L. (marijuana, hemp, cannabis; Cannabaceae) is regarded as one of the first crops humans domesticated and is primarily a dioecious diploid annual species (2*n* = 20) cultivated for fiber, oil, seed, and its medicinal and psychoactive properties (Hillig, 2005). The main pharmaceutical and psychoactive compounds are cannabinoids that accumulate in trichomes produced primarily on the floral tissues of female plants (van Bakel et al., 2011). To date, 177 cannabinoids have been identified and described, with the two most naturally abundant, well‐studied, and sought after being (−)‐*trans*‐Δ^9^–tetrahydrocannabinol (THC) and cannabidiol (CBD) (Hanuš & Hod, 2020; Hurgobin et al., 2020). These compounds are under ongoing research for their therapeutic properties. Several small studies on model organisms have investigated the use of cannabinoids in the treatment of sclerosis and Alzheimer's disease (Devinsky et al., 2014; van Amerongen et al., 2018; Watt & Karl, 2017). Additionally, cannabis produces hundreds of other bioactive compounds, such as terpenes or terpenoids and flavonoids, which are also associated with therapeutic properties (Cox‐Georgian et al., 2019). Cannabinoids, terpenes and other secondary metabolites are produced in the capitate stalked glandular trichomes, and minor variations in concentrations of each may result in distinct therapeutic effects (Hurgobin et al., 2020).

The polyketide, cannabinoid, and methylerythritol phosphate pathways are responsible for the creation of the cannabinoids (i.e., THCA, CBDA, CBCA, CBGA) and the monoterpene, mevalonate (MEV), sesquiterpene and methylerythritol phosphate pathways produce several different terpenes. The medicinal effects of cannabis plants depend on the relative concentration of these compounds and are often classified into three main categories based on the THC/CBD ratio. Type I plants express a well over 1:1 THC/CBD ratio, Type II plants have moderate amounts with a near equal proportion, and Type III plants contain a less than 1:1 ratio (Hurgobin et al., 2020). However, it should be noted that this is an oversimplification of the chemical diversity found within cannabis and that each plant has a unique chemical fingerprint that may impact its biological activity.

Clonal propagation is the primary method used when cultivating Type I & II cannabis plants for medicinal or recreational use to ensure genetic and phenotypic uniformity (McKernan et al., 2020). To achieve this, mother plants are established from elite seedlings that have been selected largely based on their specific chemical profile. Mother plants are capable of supplying hundreds or thousands of cuttings, usually taken from the apical region of the plant. The mother plants are maintained in an indefinite vegetative state for many years using a constant long‐day photoperiod (18:6 hr) and are occasionally replaced using a clonal propagule taken from themselves.

Vegetative propagation is used in many other domesticated plants to maintain valuable genotypes, including banana (*Musa acuminata* Colla), potato (*Solanum tuberosum* L.), grapevine (*Vitis vinifera* L.), hop (*Humulus lupulus* L.), and coffee (*Coffea arabica* L.) (Carrier et al., 2012; McKey et al., 2010). Theoretically, clones produce plants that are genetically identical and phenotypically similar to the parent stock. However, cannabis producers have observed a decline in the quality of clones taken from a mother plant, usually resulting in reduced cannabinoid production and plant vigor (cannabis growers, personal communication, 2020). While no peer‐ reviewed study has investigated this in cannabis, it has been discussed widely in the gray literature (Burnstein, 2019), and this is a well‐known phenomenon in other species that demonstrate a decline in plant vigor during extended periods of vegetative propagation (Muller, 1932, 1964).

**Core Ideas**

- Whole‐genome sequencing reveals genetic diversity within a single cannabis plant.
- The genetic mosaicism hypothesis applies to cannabis.
- Highest number of unique variants were found from the top tissue sample.
- Replacing mother plants from middle region clones may minimize accumulating mutations.

Muller's ratchet, a term first proposed by Felsenstein (1974), is a theory developed to explain this phenomenon and suggests that species accumulate irreversible somatic mutations in the absence of sexual recombination (Muller, 1932; Govindaraju et al., 2020). Furthermore, since the majority of random mutations are deleterious, the long‐term effect of their accumulation is a decline in plant vigor similar to what has been observed in cannabis. While somatic mutations have also positively impacted agriculture by producing unique clonal varieties of apple (*Malus domestica* Borkh.), *Citrus* spp., and grapevine derived by propagating genetically diverse bud sports, the phenomenon is problematic for the long‐term genetic preservation of elite individuals. Whether somatic mutations are beneficial or deleterious, it is well‐known that they occur and can lead to genetic diversity even within a single plant.

The source of this diversity lies in the nature of plant growth and development, in which meristematic regions grow and develop independently from one another. As they grow, each accumulates a unique set of somatic mutations that leads to genetic diversity within a plant. This phenomenon has been under investigation since the 1970s, where isozyme analysis was initially used to demonstrate genetic variation within single trees (Marshall & Allard, 1970). More recently, DNA sequencing verified this and identified that genetic distance increases systematically throughout a tree such that the mutation load is greatest at the distal end Diwan et al., 2014; Orr et al., 2020). This phenomenon is known as the genetic mosaicism hypothesis (GMH) and states that individual plants become genetically diverse because of the accumulation of spontaneous mutations occurring randomly as independent branches grow (Gill et al., 1995). While this phenomenon is often neglected during the preservation of clonal lines, it could significantly impact long‐term genetic fidelity, especially in species with higher‐than‐average mutation rates.

In this study, we examined if the GMH applies to cannabis by sequencing the full genome of three samples taken from different locations within a single mother plant. These data were used to identify nucleotide variants within the plant using bioinformatics tools and assess the degree of variation within the plant. We then calculated the unique and shared variants among samples. Lastly, we investigated the potential impact of the nucleotide variants in critical cannabinoid and terpene synthesis genes. Overall, this study confirms a significant degree of genetic variability within a single cannabis plant and raises concerns about long‐term genetic preservation using clonal propagation.

## 2 MATERIALS AND METHODS

### 2.1 Plant material

**FIGURE 1:**
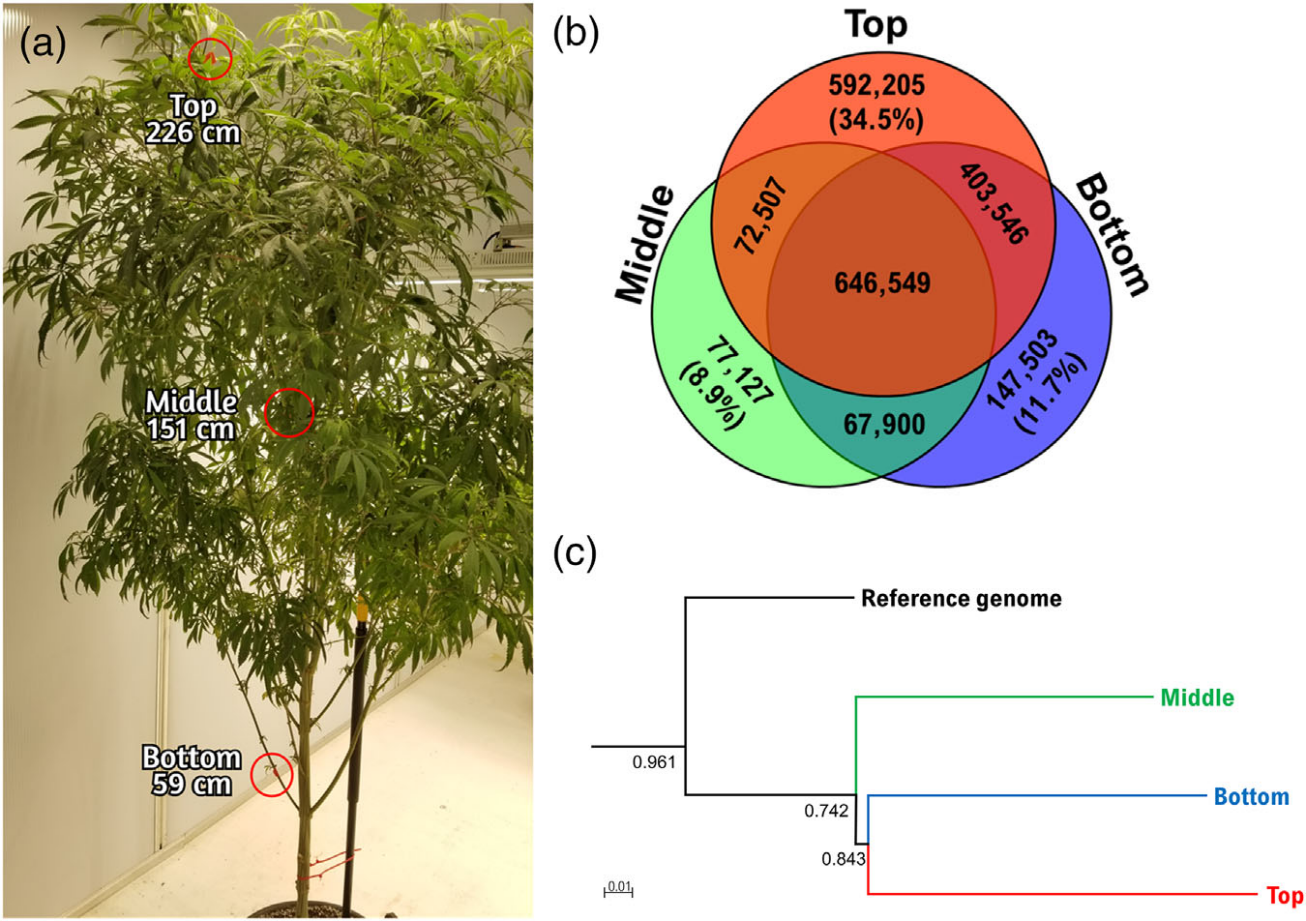
Cannabis mother plant with bioinformatic analysis. (a) ‘Honey Banana’, a mother plant (age ∼1.5 yr), exhibited incredible growth, vigor, and excellent traits, making this an ideal cultivar for this study. Three stem samples were taken from the top (226 cm), middle (151 cm), and bottom (59 cm) and had their genomes fully sequenced. (b) Venn diagram displaying the proportions of alleles shared among or exclusive to the three samples compared with the outgroup. (c) A phylogenic tree created using a genetic distance approach and the neighbor‐joining method demonstrates that the top and bottom are more similar by sharing a common node while the middle has a separate branch

A high‐THCA mother plant of cannabis cultivar Honey Banana (15–20% THC; <1% CBD) was grown indoors at BrantMed Inc. in Ontario, Canada. The plant was grown in nutrient‐rich soil with regular feeding of a complete nutrient solution developed for vegetative growth adjusted to ∼6.5 pH. The proprietary nutrient solution was relatively high in nitrogen, with moderate phosphorous levels, potassium (N‐P‐K), and micronutrients, administered every 3–5 d as needed. The plant was grown from seed and transplanted to larger pots as needed until reaching the 76‐L (20 gallon) pot, where it remained until this study was conducted. The environmental conditions were maintained at 20–25 °C and 55–65% relative humidity using BrantMed Inc LED lighting (Grow Light E1‐300W) under long‐day photoperiods at an 18:6 hr light/dark cycle to maintain the mother plant in an indefinitely vegetative state. These broad white‐spectrum lights (4.2% red 650–670 nm) with 300 W provided a photosynthesis photon flux of >440 μmol s^−1^ and came with a PAR photon efficacy of 2.2 μmol J^−1^. Samples were removed when the plant had reached an age of ∼1.5 yr. We isolated ∼2.5 cm of fresh stem tissue from three locations at 59, 151, and 226 cm, representing the bottom, middle, and top samples, respectively (Figure 1a). The samples were frozen and stored in a freezer until DNA extraction.

### 2.2 DNA extraction and whole‐genome resequencing

Frozen stem tissues were ground using a Qiagen TissueLyser. DNA was extracted from ∼100 mg of ground tissue using the Qiagen Plant DNeasy Mini Kit according to the manufacturer's protocol. DNA was quantified on a NanoDrop spectrophotometer and a Qubit fluorometer. Illumina paired‐end libraries were constructed for three DNA samples using the Illumina Tru‐seq DNA Library Prep Kit following the manufacturer's instructions. The quality of the DNA library was verified on an Agilent Bioanalyzer with a high‐sensitivity DNA chip. The sequencing was performed on an Illumina NovaSeq 6000 platform at the McGill University‐Génome Québec Innovation Center in Montreal, QC, Canada, generating >1 billion 150‐bp paired‐end reads to provide >50× death of coverage by sequencing reads against the mapped the public domain cannabis reference genome (Grassa et al., 2021). A second whole‐genome resequencing was carried out using similar protocols and procedures as previously mentioned onto the original frozen tissue used for the top sample. The Illumina NovaSeq generated 172 million reads with a depth of coverage of 32×.

### 2.3 Bioinformatic data analysis

Illumina paired‐end reads were processed using Fast‐WGS bioinformatics pipeline (Torkamaneh et al., 2018). In summary, the reads were mapped against the cs10 v2.0 cannabis reference genome (GenBank Accession No. GCA_900626175.2; Grassa et al., 2021) using BWA‐MEM (v0.7.17) (Li, 2013). This cannabis reference was chosen as it is currently the most complete reference and has been proposed to be the cannabis reference for genomics by the International Cannabis Genomics Research Consortium (Hurgobin et al., 2020). The nucleotide variants were called using Platypus (v0.8.1.1 using Python 2.7) (Rimmer et al., 2014). Detailed arguments and options used in this pipeline are available on Bitbucket (https://bitbucket.org/jerlar73/fast‐wgs/src/master/. In general, we removed variants if (a) they had more than two alleles, (b) an allele was not supported by reads on both strands, (c) the overall quality score was <32, (d) the mapping quality score was <20, (e) read depth (minimum number of reads) was <10, and (f) the number of reads supporting variant (minimum number of reads containing variants) was <10. Functional annotation of nucleotide variation was performed by SnpEFF and SnpSift (v5.0e) (Cingolani et al., 2012) using a customized reference built using the cannabis reference genome annotation file (GFF format) downloaded from NCBI (GCA 900626175.2) using default options. To obtain the description of genes with a large impact, we used the NCBI's protein table for cannabis database. The gene ontology analysis was done using the singular enrichment analysis method implemented in agri‐GO (v1.2) (Du et al., 2010).

### 2.4 Diversity, LD and clustering analysis

Nucleotide diversity (π) was calculated using –window‐pi option in VCFtools (v0.1.12b) (Danecek et al., 2011) with a window of 20,000 bp on the full dataset. An average π across all windowed calculations was used to obtain a genome‐wide average π. A neighbor‐joining phylogenetic tree was constructed using the entire dataset in Tassel (v5.0) (Bradbury et al., 2007) and the reference genome has been used as an outgroup. The reliability of clusters was assessed by bootstrapping (1000 replicates) in MEGA7 (Kumar et al., 2016) with a significant threshold of 70%. The linkage disequilibrium (LD) decay was determined with PopLDdecay (v3.40 Beta) (Zhang et al., 2019) using a VCF file containing all single‐nucleotide polymorphisms (SNPs) for all three samples. IGV (v2.8) (Robinson et al., 2011) was used to display the distribution of variants within the three samples, which was produced with the indexed version of the VCF file.

## 3 RESULTS

### 3.1 Deep full genome sequencing of bottom, middle, and top regions from a mother plant

We performed deep whole‐genome sequencing (WGS) on three samples taken from the stems located at 59, 151, and 226 cm from the top of the pot of a 2.4‐m tall, 1.5‐yr‐old mother plant (Figure 1a). This mother plant was a clonally propagated seedling selection from cannabis cultivar Honey Banana plant (BrantMed Inc.), which is a high THC type‐I plant. In total, we generated >1 billion 150‐bp paired‐end reads using an Illumina Novaseq 6000 technology (Table 1). This represents an average 58× depth of coverage across the three samples. These reads were mapped against the public cannabis reference genome (cs10 v.2.0; GenBank Accession No. GCA_900626175.2) with a mapping success rate of >93% (Table 1), thus covering >97% of the cs10 v.2.0 genome sequence (Grassa et al., 2021). Additionally, the reference genome functioned as an outgroup in which we could detect the shared and distinct allelic states from the samples. Using the Fast‐WGS (Torkamaneh et al., 2018) bioinformatics pipeline, we detected 1.3, 0.9, and 1.7 million nucleotide variants (i.e. single‐ and multiple nucleotide variants and small insertions–deletions) in the samples derived from the bottom, middle, and top of the mother plant, respectively. Initially, 6.4 million nucleotide variants were detected, but because of the high level of heterozygosity discovered, we employed a filter to both the minimum number of reads and the minimum number of reads containing variants to >10. Altogether, over 3.8 million variants compared with the outgroup were identified with a transition/transversion ratio of 1.9. As detailed in Table 1, the top of the plant had the most variants, followed by the bottom, while the middle had the fewest, demonstrating intraplant genetic diversity.

**TABLE 1:** Statistics related to the whole‐genome sequencing of cannabis samples

Sample	No. of reads	Depth of coverage	Mapping rate	SNV^a^	MNV^b^	INS^c^	DEL^d^	Total^e^
	M	*x*	%					
Bottom	362	62	94.1	966,945	104,519	95,910	98,124	1,265,498
Middle	314	54	93.8	670,974	63,953	64,168	64,988	864,083
Top	336	58	93.9	1,290,497	154,789	133,460	136,061	1,714,807

^a^ SNV, single‐nucleotide variant.

^b^ MNV, multiple‐nucleotide variant.

^c^ INS, small insertion.

^d^ DEL, small deletions.

^e^ Number of variants (allele ≠ outgroup) found within the bottom, middle, and top samples compared with the outgroup with minNR >10 and minNV >10.

### 3.2 Variant calling quality assessment

To assess the quality of genotype calls, a second run of WGS was conducted on a new DNA library extracted, using similar protocols and procedures, from the original tissue from the top. In total, we generated 172 million 150‐bp paired‐end reads using an Illumina Novaseq 6000 technology, representing an average of 32× depth of coverage across the cannabis genome. Using the Fast‐WGS (Torkamaneh et al., 2018) bioinformatics pipeline and applying a set of supervised filters to exclude low‐quality variant calls (details in Materials and Methods section), we identified 1.7 million nucleotide variants with a 99.97% agreement in variant calling with the first experiment (Supplemental Figure S1). This analysis suggests that an extensive and highly accurate set of nucleotide variants were obtained in this study.

### 3.3 Mutational variation detected inside a singular cannabis mother plant

For further analysis, the catalogues of detected variants for each sample (i.e. bottom, middle, and top) were compared with assess the variants that were shared and those that were only found in each individual sample. Of the 3.4 million variant sites identified, the top, middle, and bottom samples shared a common allele that differed from the outgroup in 38, 75, and 51% of cases, respectively. As shown in Figure 1b, the bottom and top samples shared the greatest number of variants (403,000), while the top and middle shared only 72,000 variants. The lowest overlap was observed between the bottom and middle, 67,000. Most interestingly, the top sample contained the most unique variants at 34% (592,000), followed by the bottom with 12% (147,000) and the middle with 9% (77,000). The most intriguing aspect of the result is that the top sample contained the most de novo (new) mutations. Overall, we document a very high rate of somatic mutations among all samples taken from a cannabis mother plant during vegetative growth.

According to the π statistic, the nucleotide diversity within three samples from a single plant was π = 6.0 × 10^−4^. To explore the genetic similarity between samples using nucleotide variants, we constructed a phylogenic tree using a genetic distance approach and the neighbor‐joining method with repetition 1,000× bootstrap test (Figure 1c) and used the reference genome as an outgroup. This shows two main branches, an individual middle branch as well as a bottom and top branch as the second since they share a node. Within a single plant, we also observed a rapidly declining LD (Supplemental Figure S2). The LD was seen to decay to its half in only a few kilobase pairs.

Finally, based on a visualization approach using IGV (Robinson et al., 2011), we determined if the variants were clustered in specific regions of the genome. As can be seen from Supplemental Figure S3, there were certain areas in the genome that contained fewer mutations and others where elevated levels of mutations emerged. Specifically, chromosomes 1, 5, 8, and X had an apparent increased count, while chromosomes 2, 3, 4, 6, 7, and 9 showed fewer. Furthermore, an intriguing observation was that chromosomes with more mutations also had higher levels of mutations in euchromatin regions (i.e., gene‐rich). Altogether, clusters of mutations appeared across the genome and, as a result, could indicate mutational hotspots exist within the cannabis genome.

### 3.4 Functional impacts on the genome from mutations are divergent depending on the location of the plant

**FIGURE 2:**
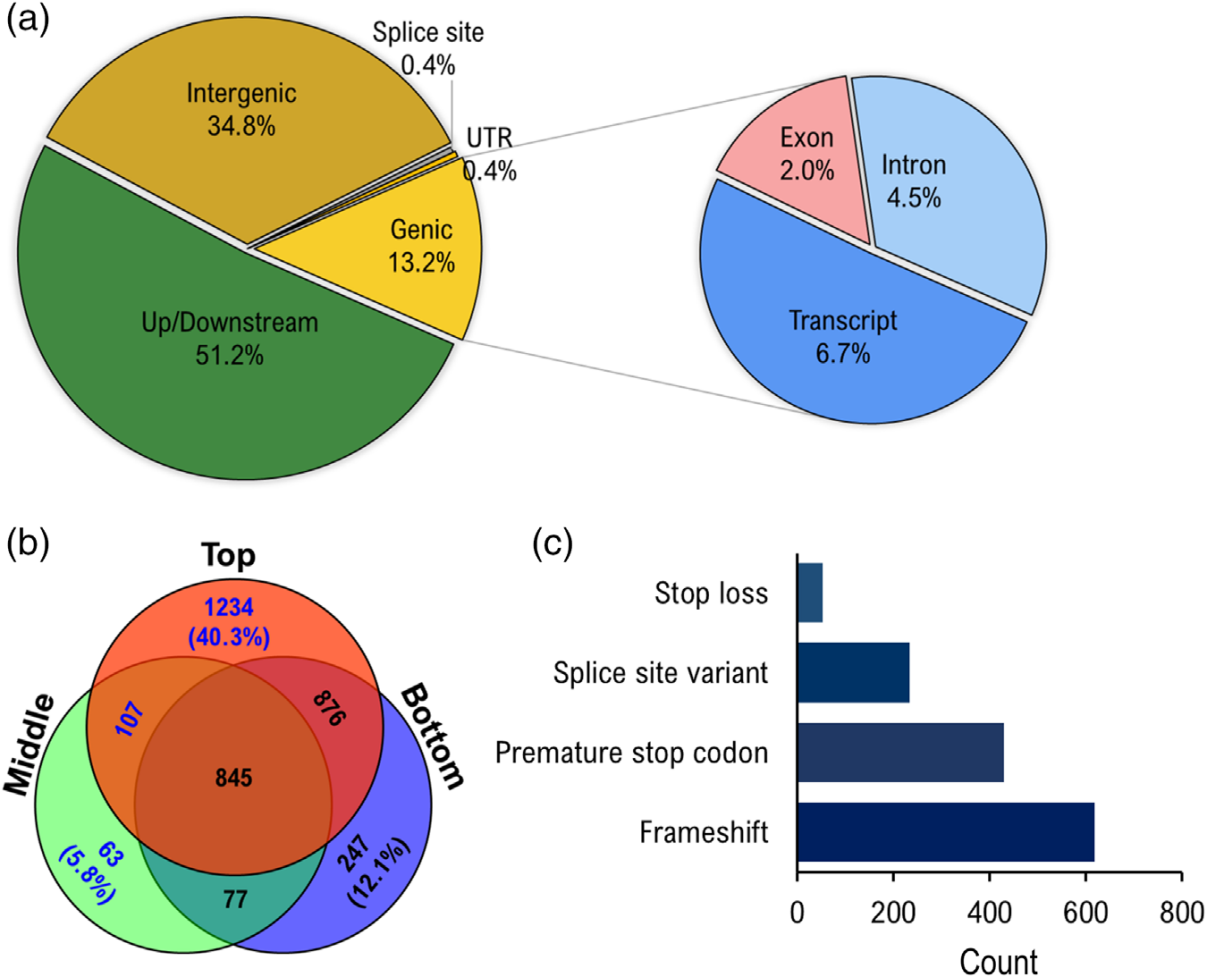
Analysis of the location and impact of nucleotide variants. (a) A pie graph visualization displays the percentages from the five main categories of found variants (intergenic, up‐ and downstream, genic, untranslated regions [UTR], and splice site), while a secondary pie graph reveals the breakdown of the genic category (transcript, exon, and intron). (b) A Venn diagram showing the high‐impact variants throughout and between the top, middle, and bottom with the blue text highlighting de novo variants. (c) High‐impact mutations were organized into four categories and represented frameshift (46%), premature stop codon (32%), splice site (17%), and stop loss (4%) mutations

To explore the potential functional impact of the mutations, we classified polymorphic variants into five categories based on their localization and identified the putative impact of the mutations. As can be seen in Figure 2a, more than half (51%) of the variants were in up‐ and downstream regions, hence in close proximity of genes (5 kb before and after gene) and the other 49% of the variants were located in intergenic regions (35%), genic regions (13%), splice sites (0.4%), and untranslated regions (0.4%). Additionally, the genic category consists of exons, introns, and transcriptional variants at 2, 4.5, and 6.7%, respectively. From a functional standpoint, we were particularly interested in the subset of mutations predicted to have a large impact. Therefore, we explored the category of the high‐impact mutations (i.e., variants which are predicted to have a disruptive effect on the protein, probably leading to protein truncation, loss of function, or triggering nonsense‐mediated decay). Figure 2b presents the unique and shared high‐impact mutations from the bottom, middle, and top samples. This information follows a similar pattern as seen for entire variants (Figure 1b), where the percentage of variants are similar. The number of shared high‐impact mutations between all samples was 845, corresponding to 41, 77, and 28% of the total high‐impact mutations for the bottom, middle, and top. The top sample had the most unique high‐impact mutations with 1,234 (40%), next the bottom with 247 (12%), and lastly, the middle with 63 (6%). The most intriguing was middle and top because there was a large difference between the unique variants (1,200) (Figure 2b). Also, they shared 952, with 107 exclusively together, representing 31% of the top and 87% of the middle total high‐impact mutations. To provide a more relevant perspective, we mainly focused on the high‐impact mutations from the middle and top samples as they are new mutations, and ultimately, they might show a different phenotype compared with the original plant (i.e., bottom). In total, 1,333 high‐impact mutations were divided into four categories and represented frameshift (46%), premature stop codon (32%), splice site (17%), and stop loss (4%) mutations (Figure 2c). These results show that a large number of high‐impact mutations (>1,000) arise as the cannabis mother plant grows and is maintained for a long time and, as a consequence, potentially severely affects the function of important genes. Owing to the lack of any significant enrichment in terms of gene ontology annotation, we investigated the functional impact of these mutations in the important cannabinoid and terpene production related genes individually using public databases.

### 3.5 Properties of novel mutations on cannabinoid and terpene pathways genes

To focus on the potential impact of these mutations more specifically on secondary metabolite production, we studied the functional impact of mutations that occurred in or near the necessary cannabinoid and terpene pathway genes. We determined which enzymes were required to create essential chemical compounds and which chromosome they can be found on the cs10 v.2.0 reference genome (Grassa et al., 2021) for both cannabinoid and terpene pathways from public databases (Table 2). As seen from Table 2, we calculated and predicted the number and type of mutations, nucleotide diversity (π) in a 20‐kb window encompassing these genes, and the contrast of the π in these genic regions compared with the genome‐wide average. Analysis of the prediction of the functional impact of the mutations determined that none of these genes contained a high‐impact mutation and recorded all observed variants as modifier. The ratio of the gene to genome‐wide π allowed us to provide a sense of potentially conserve or somatic mutation prone genes. The most notable genes that undergo somatic mutation were ones with ratio values >2.0, which includes OLS, CBDAS, HMGR2, and CsTPS9FN, with values of 2.08, 4.35, 2.95, and 2.50, respectively. Of the remaining 40 genes, 32 were under 2.0, and the other eight are missing data as a result of no data found on the NCBI's cannabis protein table. Overall, four genes are noteworthy because the number of variants emerging and seem to be more prone to somatic mutations than others, but this will require additional research to determine if this is a common trend.

**TABLE 2:** Variants in cannabinoid and terpene pathway genes

Abbreviation	Name	Chromosome	Reference	No. of variants^a^	Gene π^b^	Related to avg.^c^
Polyketide pathway						
HCS/AAE1	Hexanoyl‐CoA synthetase 1	3	Stout et al., 2012	10	2.86 × 10^−4^	0.47
OLS	Olivetol synthase	8	Taura et al., 2009	44	1.26 × 10^−3^	2.08
OAC	Olivetolic acid cyclase	9	Gagne et al., 2012	28	8.14 × 10^−4^	1.35
Cannabinoid pathway						
CBGAS	Cannabigerolic acid synthase	X	Page & Boubakir, 2014	31	8.95 × 10^−4^	1.48
THCAS	Inactive tetrahydrocannabinolic acid synthase	7	Sirikantaramas et al., 2004	2	5.71 × 10^−5^	0.09
CBDAS	Cannabidiolic acid synthase	7	Taura et al., 2007	92	2.63 × 10^−3^	4.35
CBCAS	Cannabichromosomeomenic acid synthase	7	Page & Stout, 2019	2	5.71 × 10^−5^	0.09
MEP pathway						
DXS1	DXP synthase	9	Booth et al., 2017	19	5.57 × 10^−4^	0.92
DXS2	DXP synthase	4	Booth et al., 2017	13	3.71 × 10^−4^	0.61
DXR	DXP reductoisomerase	3	Booth et al., 2017	0	0.0	0.00
MCT	MEP cytidylyltransferase	4	Booth et al., 2017	29	8.29 × 10^−4^	1.37
CMK	CDP‐ME kinase	2	Booth et al., 2017	1	2.86 × 10^−5^	0.05
MDS	MECDP‐synthase	5	van Bakel et al., 2011	16	4.71 × 10^−4^	0.78
HDS	1‐hydroxy‐2‐methyl‐2‐(E)‐butenyl 4‐diphosphate synthase	2	Booth et al., 2017	1	2.86 × 10^−5^	0.05
HDR	Hydroxymethylbutenyl diphosphate reductase	X	Booth et al., 2017	11	3.14 × 10^−4^	0.52
Monoterpene pathway						
GPPS.lsu	Geranyl pyrophosphate synthase large subunit	4	Booth et al., 2017	8	2.43 × 10^−4^	0.4
GPPS.ssu1	Geranyl pyrophosphate synthase small subunit 1	6	Booth et al., 2017	4	1.29 × 10^−4^	0.21
GPPS.ssu2	Geranyl pyrophosphate synthase small subunit 2	ND^d^	Booth et al., 2017	–	–	–
CsTPS1SK/CsTPS14CT	(−)‐limonene synthase	5	Günnewich et al., 2007	16	4.57 × 10^−4^	0.76
CsTPS2SK	(+)‐α‐pinene synthase	ND	Booth et al., 2017	–	–	–
CsTPS3FN/CsTPS15CT	β‐myrcene synthase	5	Booth et al., 2017	12	3.43 × 10^−4^	0.57
CsTPS5FN	β‐myrcene, (‐)‐α‐pinene synthase	9	Booth et al., 2017	1	2.86 × 10^−5^	0.05
CsTPS6FN	(E)‐β‐ocimene synthase	5	Booth et al., 2017	9	2.57 × 10^−4^	0.43
CsTPS13PK	(Z)‐β‐ocimene synthase	ND	Booth et al., 2017	–	–	–
CsTPS30PK	β‐myrcene synthase	5	Booth et al., 2017	9	2.57 × 10^−4^	0.43
CsTPS33PK	α‐terpinene, γ‐terpinene synthase	5	Booth et al., 2017	37	1.06 × 10^−3^	1.75
CsTPS37FN	Terpinolene synthase	ND	Livingston et al., 2020	–	–	–
CsTPS38FN	(E)‐β‐ocimene synthase	ND	Booth et al., 2017	–	–	–
MEV pathway						
HMGS	Hydroxymethylglutaryl‐CoA synthase	5	Booth et al., 2017	16	4.57 × 10^−4^	0.76
HMGR1	Hydroxy‐methylglutaryl‐CoA reductase 1	X	Booth et al., 2017	5	1.57 × 10^−4^	0.26
HMGR2	Hydroxy‐methylglutaryl‐CoA reductase 2	X	Booth et al., 2017	62	1.79 × 10^−3^	2.95
MK	Mevalonate Kinase	2	Booth et al., 2017	15	4.29 × 10^−4^	0.71
PMK	Phosphomevalonate Kinase	5	Booth et al., 2017	35	1.00 × 10^−3^	1.65
MPDC	Mevalonate diphosphate decarboxylase	1	Booth et al., 2017	15	4.29 × 10^−4^	0.71
IDI	Isopentenyl‐diphosphate delta‐isomerase	ND	Booth et al., 2017	–	–	–
Sesquiterpene pathway						
FPPS1	Farnesyl diphosphate synthase 1	4	Booth et al., 2017	8	2.43 × 10^−4^	0.40
FPPS2	Farnesyl diphosphate synthase 2	6	Booth et al., 2017	3	8.57 × 10^−5^	0.14
CsTPS4FN	Alloaromadendrene synthase	6	Booth et al., 2017	8	2.29 × 10^−4^	0.38
CsTPS7FN	δ‐selinene synthase	ND	Booth et al., 2017	–	–	–
CsTPS8FN	γ‐eudesmol, valencene synthase	6	Booth et al., 2017	15	4.38 × 10^−4^	0.72
CsTPS9FN	β‐caryophyllene, α‐humulene synthase	6	Booth et al., 2017	53	1.51 × 10^−3^	2.50
CsTPS16CC	Germacrene B synthase	ND	Zager at al., 2019	–	–	–
CsTPS20CT	Hedycaryol synthase	6	Booth et al., 2017	38	1.10 × 10^−3^	1.82
CsTPS18VF/CsTPS19BL	Nerolidol/linalool synthase	1	Booth et al., 2017	5	1.43 × 10^−4^	0.24

^a^ Includes 10 kb before and after gene.

^b^ Window range 21 kb.

^c^ Whole‐genome nucleotide diversity = 6.0 × 10^−4^.

^d^ND, not determined in cs10 v2.0 genome.

## 4 DISCUSSION

### 4.1 Genomic diversity within cannabis

Numerous studies have investigated intraplant somatic mutations in long‐lived perennials, but few have examined this in annuals, and this research is the first to look at intraplant mutations in cannabis. Diwan et al., 2014; Plomion et al., 2018; Hanlon et al., 2019; Orr et al., 2020). Many previous studies used molecular markers to investigate and calculate mutation rates, which are prone to miss more rare mutations that WGS will capture. Some early research on intraplant genetic variation was unreliable as they used extrapolations from microsatellites or chlorophyll mutations. However, more recent research has uncovered a more comprehensive understanding because of advances and technologies becoming more accessible (Schoen & Schultz, 2019). For example, two studies using WGS on a long‐lived oak (*Quercus robur* L.) tree reported a much lower mutation rate than what was obtained from scaling up estimates from *Arabidopsis thaliana* (L.) Heynh. Based on this, they hypothesized that perennials may have a mechanism to reduce the accumulation of mutations, which is lacking in short‐lived annual species (Schmid‐Siegert et al., 2017; Plomion et al., 2018). Likewise, in mammals, it has been shown that mice have more mutations per cell division than humans, which is consistent with the hypothesis that short‐lived species have higher mutation rates (Milholland et al., 2017). Currently, somatic mutations are believed to be common in plants, but the mutation rate, distribution, morphological effects, age, or size influence and the differences between annuals and perennials remains poorly understood (Schoen & Schultz, 2019).

Naturally, cannabis is an annual species where it lives until its flowers are pollinated and seeds are produced. This all occurs during a single season that can range from a few months to closer to a year and then it naturally dies. In contrast, cannabis plants maintained as mothers are artificially maintained in a perpetual vegetative state and replaced periodically using clonal propagules that can extend their lifespan to several years or even decades. Based on Muller's ratchet, a decline in plant vigor is likely during this period because of the accumulation of deleterious mutations and the absence of recombination. The extended lifespan of mother plants significantly increases this concern because each de novo deleterious mutation bears the potential for both multiplicative and cumulative effects to genome instability, altered gene expression, molecular heterogeneity, tissue disintegration, and vulnerability to stress (Govindaraju et al., 2020). In other species, propagules that inherit deleterious mutations from a mother plant exhibited smaller leaves, reduced nutrient translocation capacity, degraded genetics, less rooting, lower plant vigor, and a decrease in growth (Orr et al., 2020; Singh et al., 2015; Wendling et al., 2014). Therefore, our results lead us to hypothesize that prolonging the lifespan of cannabis plants and constantly pruning clonal cuttings leads to the observed plant decline.

In this study, we used the reference genome (cs10 v2.0; Grassa et al., 2021) in the first step of analysis of sequencing data to accurately align sequencing reads in their corresponding genomic regions and as well as a common ancestor genome (outgroup). Thus, allowing the examination for unique genomic variation from our test genomes derived from different regions of a mother plant. Our results revealed many variants that were unique to each individual sample and thus, displayed the divergence of cell lines within an individual mother plant.

In plants, there lacks a segregated germline and instead, somatic meristem cells produce vegetative tissues until late in development, where reproductive organs are eventually formed (Watson et al., 2016). As a result, each meristematic cell line can carry over unique mutations that may have occurred during the formation of new vegetative tissues. Therefore, creating what can be viewed as a metapopulation because of each cell line accumulating a variety of mutations throughout its growth (Wang et al., 2019)

In clonal propagation, the clonal cuttings used to propagate will inherit these mutations and continue to grow with their own additional mutation accumulation. Normally, if specific branches accumulate too many harmful mutations, they could be outcompeted by more healthy and rapidly growing sections, as each branch is derived from independent meristems. This process is known as developmental selection (Cruzan et al., 2020). Each meristem will contain a variety of diverse mutations and effects, as not all mutations are equal. In the case of cannabis mother plants, they are used to propagate up to hundreds of propagules at a time and thereby would possibly include clones from branches that may already be performing poorly as a result of the accumulation of deleterious mutations.

We identified somatic mutations with deep sequencing (>50 × depth of coverage) and used systematic filtering steps to reduce errors that may have occurred from next‐generation sequencing and incorrect mapping (Ajay et al., 2011). Additionally, to assess the possibility of inaccuracies, resequencing was performed on the original top tissue using the same protocol and procedure. The comparison between these samples was used to confirm the quality of genotyping and revealed an astonishing 99.97% agreement in variant calling. Thus, indicating that errors or incorrect mapping had nearly zero effect on the callings of variants.

A recent cannabis study sequenced 40 cannabis genomes, and they reported an average of 12.8 million SNPs and insertions–deletions for dispensary‐grade cannabis (Type I and Type II plants) which equated to a variant every ∼73 bases, while our results equated to a variant every ∼425 bases (McKernan et al., 2020). Although our variants were sixfold less than the average found in the other study, partly because of the clonal origin of our samples, they still represent a substantial quantity of variants that contain the ability to interfere with the stability and quality of the plant.

### 4.2 Variants across intraplant genome

We expected to witness a systematically hierarchical nature of mutation accumulation from the bottom to the top, as was seen by Diwan et al. (2014) in their research on genome differences within Yoshino cherry (*Prunus × yedoensis* Matsum.) and Japanese beech (*Fagus crenata* Blume) trees. In this study, we witnessed a minor drop in total variants between the bottom and middle compared with the middle to the top. Although we did not observe a uniform increase, the uppermost sample was more genetically distant from the bottom than was the middle, as seen by other previous intraplant studies (Schmid‐Siegert et al., 2017; Plomion et al., 2018; Hanlon et al., 2019). Additionally, the primary analytical focus in this study was the middle and top variants because they represent de novo mutations, while the bottom section corresponds to the oldest growth tissue where, in theory, the mutations counts should be the lowest.

In the case of cannabis, mother plants are kept for prolonged periods and looked after under controlled conditions with special maintenance techniques, making their genetic lineage more complex than what has been observed in other species. One example of this, which was the method used at our growth facility, involves pruning the lower region of the plant to encourage more growth to the upper sections where they are used as propagules. This promotes more cell division at these regions and may help explain why there was not a direct relationship between tissue location and genetic divergence. In the end, it seems that the middle area of the plant is the most stable and the biologically oldest section of the plant as fewer cell divisions are occurring when compared with the top and bottom of a cannabis mother plant, but further research is needed with larger sample numbers.

While many uncertainties remain about somatic mutation in plants, our results demonstrating significant genetic variation may be explained by a few possibilities: (a) environmental factors, (b) long‐term pruning from the top, (c) difference between perennials and annuals, and (d) small sample size. Environmental factors have been well studied and are known to impact mutation rates, especially during shock or stress (Gill et al., 1995). Thus, any changes to the environment during the growth and maintenance of the mother plant, such as light or temperature stress, may have altered the mutation rates and contributed to their accumulation over time. Next, because of the nature of cannabis mother plants, they are consistently apically pruned, and clonal propagules are taken for propagation. This practice directly impacts the balance between auxin and cytokinin levels, promotes rapid growth from originally inhibited axillary bud sites, and, as a result, could impact the rate of mutations within the plant (Prusinkiewicz et al., 2009; Schaller et al., 2015). Recently, next‐generation sequencing studies identified major differences in the rate of mutations between perennials and annuals species, with perennials having a significantly lower rate than expected, suggesting they may possess a mechanism to suppress the rate (Schoen & Schultz, 2019). These findings are consistent with our results that found a relatively high number of mutations and lead us to believe that annuals contain a more severe and sporadic mutation rate such that prolonging their natural lifespan may lead to a substantial mutation load with potentially deleterious effects. Lastly, because of the expensive costs of full‐genome sequencing and intention to identify intraplant variations, only three positions (i.e., bottom, middle, and top) were sequenced with the ability to call ultra‐rare mutations. As such, it is unknown if the degree of diversity observed in this study is representative of the species. Further work is needed using larger sample sizes across multiple genotypes in different environments to gain a better understanding of this phenomenon.

### 4.3 Impact on cannabinoid and terpene synthase

We investigated the impact these mutations may have had on both the cannabinoid and terpene pathways because of their importance in medical and product quality (Hanuš & Hod, 2020; Singh et al., 2020). First, we examined mutations categorized as high‐impact from analysis using SnpEff (Cingolani et al., 2012). These mutations are known to have a large impact on protein production, protein truncation, loss of function, or trigger nonsense‐mediated decay (Cingolani et al., 2012). Although we did not identify any high‐impact mutations in the cannabinoid and terpene synthase genes, the analysis revealed a similar arrangement of the distribution of mutations from the total number of variants. Interestingly, the top sample contained an even larger total percentage by ∼6%, while the middle had 3% less and the bottom <1% difference. This further supported the notion that apical regions are more genetically distant than the basal regions.

Furthermore, we examined mutations in these pathway genes that fell under the categories of moderate, low, or modifier, which relates to nondisruptive changes to protein effectiveness, mostly harmless or unlikely to change protein performance, and noncoding variants where predictions are difficult or there is no evidence of impact, respectively. Our analysis revealed that all discovered variants fell under the modifier category and encouraged the assessment of nucleotide diversity (π) with a 20‐kb scope of critical genes for both pathways. Most of the 44 genes investigated had a similar or lower value than the average nucleotide diversity (π); however, four major genes had values over double. Two of these are a part of terpene production (HMGR2 and CsTPS9FN), and the other two are involved in the cannabinoid pathway (OLS and CBDAS). The role of HMGR2 is to convert acetyl‐CoA into mevalonic acid, which undergoes a few more steps to produce the synthesis of both γ‐eudesmol or β‐caryophyllene terpenes. CsTPS9FN is the last enzyme necessary to produce the β‐caryophyllene terpene. OLS and CBDAS were particularly intriguing because OLS is an essential enzyme for the whole cannabinoid pathway. It converts hexanoyl‐CoA into olivetolic acid, which converts into CBGA, a precursor to many well‐known cannabinoids (i.e., THCA, CBDA, CBCA). CBDAS is the last enzyme required to convert CBGA into CBDA, and this is especially important as it is the prominent cannabinoid used to treat various health concerns (Maroon & Bost, 2018). Both critical enzymes revealed a more than double nucleotide diversity (π) above average and, as a result, could indicate initial signs of decay in the cannabinoid pathway.

Mother plants are usually selected through large‐scale, costly screening programs and marketed as strains with unique properties. The alteration of genes over time represents a significant challenge in long‐term batch‐to‐batch consistency. Also, cannabis used for medicinal purposes must ensure a consistent product that provides the appropriate properties and quality necessary for treatments. Thus, a greater insight into somatic mutations may enable new or superior procedures to assist the preservation of elite cultivars in clonally propagated plants. Overall, our research highlights an important phenomenon related to maintaining elite genetics and could provide an underlying mechanism for the decay of cannabinoids and plant vigor that has been anecdotally observed (Cannabis growers, personal communication, 2020).

## 5 CONCLUSION

The findings in this study demonstrate that genetic diversity exists with a single cannabis plant and the genetic mosaicism hypothesis applies to cannabis. This study is the first to investigate the existence of this phenomenon in cannabis plants and the potential consequences of accumulating somatic mutations in an artificially prolonged annual species. As cannabis normally lives for ∼3–6 mo, this process likely enables an unknown but manageable amount of somatic mutations to accumulate. Currently, somatic mutations in plants have many uncertainties remaining, but as a result of modern genetic technologies and more affordable WGS, there have been more contributions with higher degrees of accuracy and precision on this topic. From a practical standpoint, this significantly benefits the cannabis industry as understanding this phenomenon will help establish best practices for maintaining mother plants to minimize, slow, or prevent the accumulation of mutations. Based on these data, we advocate replacing mother plants using cuttings from the basal portion of the plant and discourage excessively extending the life of a mother plant. Additionally, important genetics should be preserved using cryopreservation techniques where the original genetic profile can be maintained and accessed indefinitely (Uchendu et al., 2019). The research here provides a concrete basis for cannabis mutation research. However, the current study lacked different cultivars, generational data, mutation rates and multiple biological replicates. Thus, future research will be necessary to enhance and solidify our understanding of somatic mutations and the mutagenic potential within a cannabis mother plant.

## AVAILABILITY OF DATA AND MATERIALS

The dataset used in this study is available at https://figshare.com/projects/Accumulation_of_somatic_mutations_leads_to_genetic_mosaicism_in_Cannabis/118161.

## AUTHOR CONTRIBUTIONS

Kristian Adamek: Data curation; Formal analysis; Investigation; Methodology; Supervision; Validation; Visualization; Writing‐original draft; Writing‐review & editing. Andrew Maxwell Phineas Jones: Conceptualization; Funding acquisition; Investigation; Methodology; Project administration; Resources; Supervision; Validation; Visualization; Writing‐review & editing. Davoud Torkamaneh: Conceptualization; Data curation; Formal analysis; Funding acquisition; Investigation; Methodology; Project administration; Resources; Software; Supervision; Validation; Visualization; Writing‐review & editing

## CONFLICT OF INTEREST

The authors declare that they have no competing interests.

## Supporting information


**Supplemental Figure S1**. Comparison of nucleotide variants from two separate WGS of the top sample.
**Supplemental Figure S2**. LD decay graph revealing a rapid decay in only a few kb.
**Supplemental Figure S3**. Visual representation of nucleotide variants distributed throughout the chromosomes.
