# Supplementary material for: Accumulation of somatic mutations leads to genetic mosaicism in cannabis
Source: Plant Genome. 2021 Nov 22;15(1):e20169. doi: 10.1002/tpg2.20169 (PMC12807173; doi:10.1002/tpg2.20169)
Supplement: Supplementary file 1 [file TPG2-15-e20169-s001.docx]

**Accumulation of somatic mutations leads to genetic** **mosaicism in Cannabis**

Kristian Adamek^1^, Andrew Maxwell Phineas Jones^1^, Davoud Torkamaneh^2*^

^1^ Department of Plant Agriculture, University of Guelph, Guelph, N1G 2W1, ON, Canada

^2^ Département de Phytologie, Université Laval, Québec, G1V 0A6, QC, Canada

* Correspondence: davoud.torkamaneh.1@ulaval.ca

**Supplemental Materials**

**Supplemental Figure S1.** Comparison of nucleotide variants called from the first and second runs of WGS on the original top tissue with a near identical agreement in variant calling.

**Supplemental Figure S2.** LD decay graph that shows a rapid decay to half in merely a few kb.

**Supplemental Figure S3.** A visual representing the distribution of nucleotide diversity through the full genome from the bottom, middle and top samples. Chromosomes 1, 5, 8 and X are under a heavier burden of nucleotide variants.
